# Avocado Consumption Patterns and Nutrient Contribution in the US: National Health and Nutrition Examination Survey 2017–March 2020 and August 2021–August 2023

**DOI:** 10.3390/nu18030449

**Published:** 2026-01-29

**Authors:** Feon W. Cheng, Suzanne Morton, Megan A. McCrory, Alanna J. Moshfegh, Nikki A. Ford

**Affiliations:** 1 Avocado Nutrition Center, 25212 Marguerite Pkwy Ste. 250, Mission Viejo, CA 92692, USA; nikki@hassavocadoboard.com; 2 Food Surveys Research Group, Beltsville Human Nutrition Research Center, Agricultural Research Service, United States Department of Agriculture, Beltsville, MD 20705, USA; suzanne.morton@usda.gov (S.M.); alanna.moshfegh@usda.gov (A.J.M.); 3 American Society for Nutrition, 2440 Research Boulevard, Suite 430, Rockville, MD 20850, USA; 4 Department of Health Sciences, Sargent College of Health and Rehabilitation Sciences, Boston University, Boston, MA 02215, USA; mamccr@bu.edu

**Keywords:** avocado, diet, nutrient

## Abstract

Background/Objectives: Avocados are nutrient-dense fruits rich in monounsaturated fats, fiber, and key micronutrients. Although avocado purchases increased in recent years, comprehensive national data on consumption patterns remains limited. Thus, this study aimed to characterize the prevalence, quantity, and context of avocado intake among the US population and to evaluate its contribution to daily nutrient intake. Methods: Day 1 24-h dietary recall data were analyzed from 19,086 participants aged ≥1 year in NHANES 2017–March 2020 and August 2021–August 2023. Avocado intake was identified using consumption data coded as avocado, avocado for use on a sandwich, guacamole, and guacamole with tomatoes. Weighted estimates described consumption prevalence, amount, form, self-selected eating occasion, location, and source. Nutrient contributions from avocado were calculated overall and by sex, age, and race/ethnicity. Results: 5.8% of participants consumed avocado. Prevalence was highest among women (6.9%), adults aged 19–50 years (7.4%), and Hispanic individuals (9.1%). Among consumers, the mean intake was 67.0 g/day. Plain avocado was the most common form (54.8%), and most intake occurred at dinner (43.4%) and at home (67.7%). Avocado was an important contributor to the mean daily intake among consumers for beta-cryptoxanthin (33.4%), alpha-carotene (27.0%), dietary fiber (19.9%), monounsaturated fatty acids (19.7%), and other nutrients. Conclusions: Although avocado consumption remains relatively modest, it contributes meaningfully to nutrient intake among avocado consumers. These findings provide descriptive insight into avocado consumption patterns and nutrient contributions in the U.S. population.

## 1. Introduction

Avocados (Persea americana) are nutrient-dense fruits that offer a unique combination of macronutrients and micronutrients, including monounsaturated fats, dietary fiber, potassium, magnesium, folate, and vitamins E and K [1]. Unlike most fruits, avocados are low in sugar and provide a substantial proportion of energy from unsaturated fats, a composition that has been associated with improved nutrient adequacy and favorable cardiometabolic markers in observational and clinical research [1,2,3,4,5,6,7]. As a result, avocados are nutritionally compatible with healthful dietary patterns, including Mediterranean-style and plant-based diets [8,9].

Worldwide avocado consumption has experienced sustained growth over recent decades, with total intake rising approximately threefold—from 6 billion to 18 billion pounds—between 2000 and 2020 [10]. In 2020, about one-third of global avocado consumption occurred in Asia-Oceania, Africa, and Europe, while the remaining two-thirds were consumed in the United States, Latin America, and the Caribbean. In the United States, avocado availability has increased substantially over the past two decades. According to the United States Department of Agriculture (USDA), per capita availability of avocados tripled between 2000 and 2021, exceeding 8 pounds per person annually [11]. Despite this growth, comprehensive data on avocado consumption behaviors, including typical amounts consumed, eating contexts, and nutrient contributions, remain limited.

Two prior analyses using earlier National Health and Nutrition Examination Survey (NHANES) cycles have examined avocado consumption in relation to overall diet quality and selected health outcomes. These studies primarily compared avocado consumers with non-consumers and reported average intake amounts or associations with total energy and nutrient intakes and cardiometabolic markers [12,13]. However, avocado intake in these analyses was restricted to plain avocado and avocado for use on a sandwich, while other commonly consumed mixed dishes, such as guacamole-based preparations, were not included. These studies also did not comprehensively characterize avocado consumption behaviors or quantify the relative contribution of avocado to daily energy and nutrient intakes across commonly consumed forms. Furthermore, both studies were based on earlier NHANES cycles (e.g., 2001–2008 and 2001–2012) that precede recent shifts in dietary patterns, food availability, and avocado consumption trends in the United States [11]. Given the substantial changes in avocado availability and consumption over the past decade [11], updated analyses using more recent NHANES data are warranted. Moreover, a detailed examination of avocado-derived nutrient contributions—expressed using both absolute and energy-adjusted measures—can provide important context for understanding the role of avocados within contemporary dietary patterns.

Therefore, the present study used recent data from the National Health and Nutrition Examination Survey (NHANES) to examine current patterns of avocado consumption in the US population. Specifically, it (1) estimates the percentage of individuals who consumed avocado on a given day, (2) quantifies the average amount consumed and the most common forms of consumption, (3) assesses the contribution of avocado to daily nutrient intakes, including the percentage of total energy and nutrients derived from avocado and energy-adjusted intakes per 1000 kcal, and (4) describes the self-selected eating occasions, location, and source of avocado consumption. By providing a detailed characterization of avocado consumption patterns and nutrient contributions, this study extends prior NHANES-based research and offers new insight into how avocados contribute to the diets of U.S. consumers.

## 2. Materials and Methods

### 2.1. Study Sample

**Figure 1:**
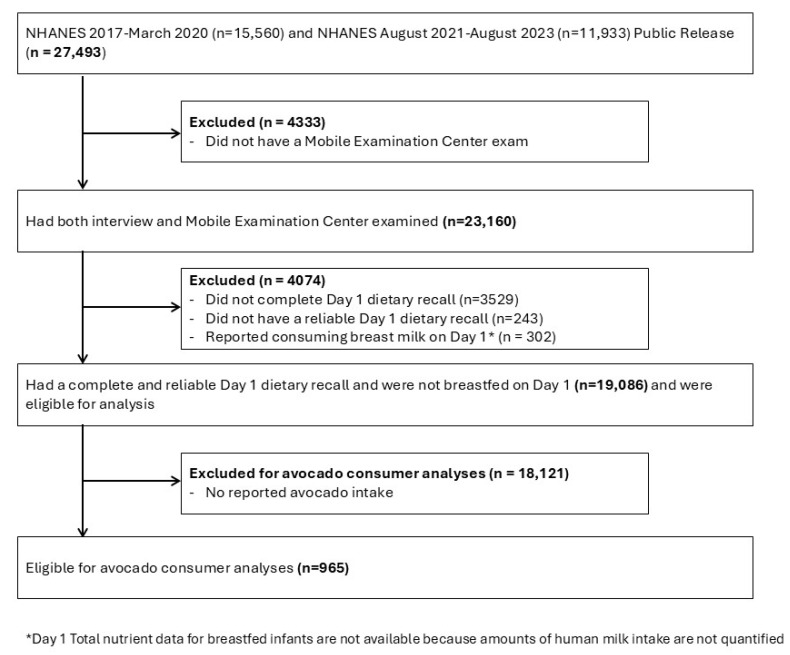
Flow chart of eligible National Health and Nutrition Examination Survey (NHANES) participants included in this analysis.

Of 27,493 NHANES participants from 2017–March 2020 (pre-pandemic) and August 2021–August 2023, 19,086 had a complete and reliable Day 1 24-h multiple pass dietary recall interviews in What We Eat in America and were not breastfed on Day 1 (Figure 1). NHANES is a publicly available dataset administered by the National Center for Health Statistics (NCHS) at the Centers for Disease Control and Prevention (CDC). It employs a complex, multistage, stratified, and clustered probability sampling design and releases data in two-year intervals [14]. Due to disruptions caused by the COVID-19 pandemic, data from 2019–2020 were combined with the 2017–2018 cycle to form the NHANES 2017–March 2020 dataset, as the 2019–2020 cycle alone was not nationally representative [14]. All NHANES participants provided informed consent, and study protocols were approved by the NCHS Ethics Review Board. As NHANES data are de-identified and publicly available, this secondary analysis was exempt from additional ethical review.

### 2.2. Dietary Assessment

Trained interviewers collected dietary data using the USDA’s Automated Multiple-Pass Method [15]. For the NHANES 2017–March 2020 dataset, two 24-h dietary recalls were conducted: the first in person at a Mobile Examination Center (MEC) and the second by telephone 3–10 days later. For the August 2021–August 2023 dataset, both recalls were conducted by telephone. The present analysis used data from the first recall to estimate average population intake on a given day. For participants younger than 12 years old or unable to self-report, a parent or proxy may have responded on their behalf. Additional details on dietary data collection are available in the NHANES Interview Procedures Manual and related publications [16,17,18].

Avocado consumption was identified using four USDA food codes from the Food and Nutrient Database for Dietary Studies (FNDDS): avocado, avocado for use on a sandwich, guacamole, and guacamole with tomatoes. Participants who reported consuming any of these items on Day 1 were classified as avocado consumers. Nutrient intakes from avocados were estimated using FNDDS [19]. Participants also reported the eating occasion, food source, and whether it was consumed at home. Day 1 dietary recall data were used because the objective of this study was to characterize avocado consumption on a given day, including the prevalence of consumption, amounts consumed, forms of intake, eating occasions, and associated nutrient contributions [20]. The first recall generally has higher response rates and data completeness than the second recall across NHANES cycles [21,22]. Because avocados are episodically consumed, analyses focused on consumers provide more meaningful insight into consumption context and nutrient contribution on days avocados are eaten.

### 2.3. Demographic Characteristics

Demographic characteristics examined were age (<19, 19–50, >50 years), sex (men and women), race/ethnicity (Hispanic, non-Hispanic White, non-Hispanic Black, and other race—including multi-racial), marital status (married/living with partner, widowed/divorced/separated, and never married), education (<9th grade or 9–11th grade, high school grad/GED or equivalent, and some college or AA degree or college graduate or above), and family income to poverty ratio (<131%, 131–350%, and >350%).

### 2.4. Statistical Analysis

All analyses accounted for the complex, multistage sampling design of NHANES and applied appropriate survey weights to generate nationally representative estimates. Descriptive statistics were used to summarize participants’ demographic characteristics. Mean avocado intake (grams), avocado-derived energy and nutrient intakes (absolute), and energy-adjusted intakes expressed per 1000 kcal were estimated. Independent sample *t*-tests and chi-square tests were employed to compare mean gram, energy, and nutrient intakes from avocado consumption, as well as the percentage contribution of avocados to total nutrient intake, across sex, age group, and race/ethnicity. Percentages of total daily nutrient intake from avocado were calculated as the proportion of each nutrient derived from avocado relative to total daily intake of that nutrient. For comparisons involving multiple subgroups, one-way analysis of variance (ANOVA) with Tukey–Kramer post hoc test was used. Eating occasion type and source of avocado consumption were summarized using avocado consumption reports on Day 1 dietary recalls to estimate weighted percentages and standard errors; because participants could report avocado consumption more than once on the same day, the number of consumption reports exceeded the number of avocado consumers. All statistical analyses were performed using SAS version 9.4 (SAS Institute Inc., Cary, NC, USA), with statistical significance defined as *p* < 0.05.

### 2.5. Protocol Registration and Checklist

The study protocol was developed and preregistered prior to data analysis on the Open Science Framework at https://osf.io/4vcbm [23] (accessed on 23 November 2024). To ensure transparency and adherence to best practices in reporting, the study followed the Strengthening the Reporting of Observational Studies in Epidemiology checklist [24].

## 3. Results

### 3.1. Percentages of Avocado Consumers

Among participants who were included in the analysis, 5.8 ± 0.3% (weighted percentage ± standard error) reported consuming avocado (Table 1). Among avocado consumers, the majority (*n* = 874) reported a single avocado consumption event on Day 1, while fewer participants reported two (*n* = 81), three (*n* = 7), four (*n* = 2), or five (*n* = 1) avocado consumption reports on the same day. The prevalence of avocado consumption varied across demographic groups (Table 1A). Adults aged 19–50 years had the highest consumption rate, while those under 19 years had the lowest. Women were more likely to consume avocado than men. By race/ethnicity, Hispanic individuals had the highest percentages of avocado consumers. Consumption was more prevalent among individuals with higher educational attainment and income levels. As shown in Table 1B, avocado consumers had higher mean total energy intake, a greater percentage of energy from total fat and a lower percentage from carbohydrates, and higher fiber intake (g/1000 kcal) compared with non-consumers.

### 3.2. Mean Intake and Most Common Forms of Avocado Consumption

Among consumers (n = 965), the average avocado intake was 67.0 ± 2.9 g per day (Table 2). Plain avocado was the most frequently consumed form, reported by 54.8% ± 1.4% of consumers, with a mean intake of 70.8 ± 3.0 g per day. Guacamole was the second form most frequently reported by consumers (24.0% ± 2.1%, 65.1 ± 7.2 g per day), followed by avocado used in sandwiches (15.0% ± 1.4%, 32.6 ± 2.2 g per day) and guacamole with tomatoes (10.3% ± 1.0%, 74.7 ± 6.1 g per day). Plain avocado was also the most consumed form across all sex, age, and racial/ethnic groups.

### 3.3. Nutrient Contributions of Avocado Consumption

Avocados contributed key nutrients to consumers’ diets (Table 2). The average intake per day included 9.6 ± 0.4 g of total fat—primarily monounsaturated fat (6.4 ± 0.3 g)—5.7 ± 0.2 g of total carbohydrates (including 4.4 ± 0.2 g of dietary fiber), and 1.3 ± 0.1 g of protein. Avocados also supplied notable amounts of various micronutrients. Energy-adjusted (per 1000 kcal) daily intakes of avocado and avocado-derived energy and nutrients did not differ by sex. By age group, individuals <19 years had higher energy-adjusted beta-carotene intake compared with those aged 19–50 years. Post hoc comparisons using the Tukey–Kramer adjustment did not identify significant pairwise differences across other age groups or race/ethnicity.

### 3.4. Percentages of Total Nutrient Intakes from Avocado Consumption

Table 3 shows the percentage contribution of avocados to mean daily nutrient intake among avocado consumers, stratified by sex, age, and race/ethnicity. On average, avocado contributed 5.3 ± 0.2% of daily energy and 11.4 ± 0.4% of daily fat intake. Among fats, monounsaturated fats had the highest contribution. The top five nutrients with the greatest percentage contributions from avocado were beta cryptoxanthin, alpha carotene, dietary fiber, monounsaturated fat, and lutein + zeaxanthin. Significant age-related differences were observed in avocado’s contribution to mean daily intakes for several nutrients. Among individuals under 19 years old, avocado contributed a higher percentage to copper, vitamin B-6, beta carotene, lutein/zeaxanthin, vitamin E and vitamin K intake compared to individuals 19–50 years old. Avocados contributed a greater percentage of daily intake of vitamin C and lutein/zeaxanthin among males than females. Post hoc comparisons using the Tukey–Kramer adjustment revealed significant pairwise differences by race/ethnicity for saturated fat, dietary fiber, potassium, and copper.

### 3.5. Eating Occasion, Location, and Sources of Avocado

**Figure 2:**
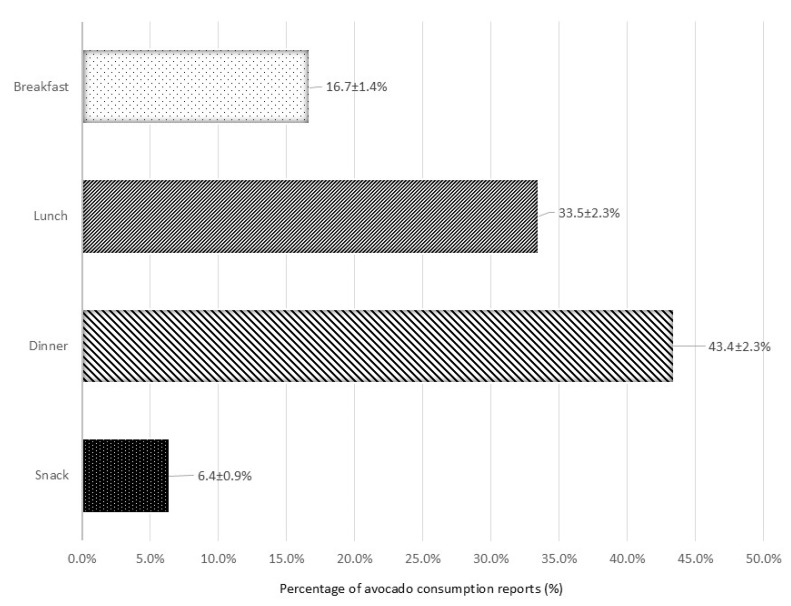
Percentage ± standard error of avocado consumption reports by eating occasion type (number of reports = 1070) on day 1 of intake in the National Health and Nutrition Examination Survey (NHANES), 2017–March 2020 (Pre-Pandemic) and August 2021–August 2023. Note that since the number of reports exceeds the number of consumers (n = 965), some consumers reported more than one instance of consumption.

**Figure 3:**
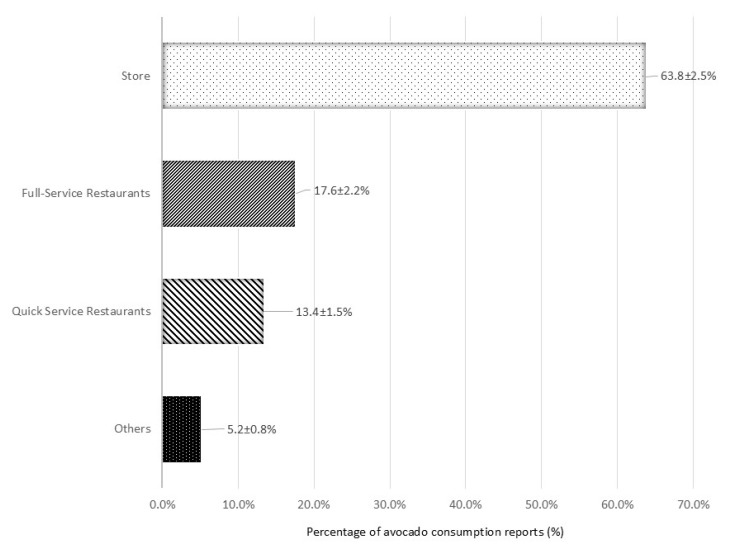
Percentage ± standard error of avocado consumption reports by source (number of reports = 1070) on day 1 of intake in the National Health and Nutrition Examination Survey (NHANES), 2017–March 2020 (Pre-Pandemic) and August 2021–August 2023. Note that since the number of reports exceeds the number of consumers (n = 965), some consumers reported more than one instance of consumption. Full-service restaurants included: restaurant with waiter/waitress, Bar/tavern/lounge, or Restaurant no additional information. Quick service restaurants included: restaurant fast food/pizza, Cafeteria NOT in a K-12 school, Sport, recreation, or entertainment facility, Street vendor, vending truck. Store included grocery/supermarket and convenience-type. Others included cafeteria in a K-12 school, child/adult care center, child/adult home care, soup kitchen/shelter/food pantry, Meals on Wheels [26] (a federally supported, community-based home-delivered meal program for older adults), community food program—other, community program no additional information, vending machine, common coffee pot or snack tray, from someone else/gift, mail order purchase, residential dining facility, grown or caught by you or someone you know, fish caught by you or someone you know, fundraiser sales, store—no additional info, and other.

About two-thirds (67.7 ± 2.4%) of avocado consumption reports occurred at home. Most avocado consumption reports occurred during dinner (43.4 ± 2.3%), followed by lunch (33.5 ± 2.3%), breakfast (16.7 ± 1.4%), and snack (6.4 ± 0.9%) (Figure 2). Over 60% of avocado consumption reports were sourced from supermarkets or convenience stores, with smaller proportions obtained from full-service and quick-service restaurants (Figure 3).

## 4. Discussion

In this nationally representative dataset of US children and adults using NHANES data from 2017–March 2020 and August 2021–August 2023, 5.8% of participants reported consuming avocado on a given day, based on Day 1 dietary recall data. This prevalence surpasses previously reported estimates from earlier NHANES cycles, which documented avocado unweighted consumption rates of about 2.0% (2001–2008) and 2.2% (2001–2012) among adults aged 19 years and older [12,13]. In the current analysis, prevalence was 7.4% among adults aged 19–50 years and 5.5% among those over 50 years. The higher prevalences observed may be partially attributable to the broader classification criteria used to define avocado consumers. Specifically, the present study incorporated four USDA food codes, whereas earlier studies [12,13] relied exclusively on a single code for plain avocado, which also captured avocado for use on a sandwich reports. When our analysis was restricted to that code alone, the prevalence was lower (3.9%), though it remained higher than prior estimates. This comparison suggests that the higher prevalence observed in the present study reflects both broader capture of avocado-containing foods and increased reporting of avocado consumption relative to earlier NHANES cycles, rather than a definitional artifact alone (Appendix A Table A1). Formal trend analyses across multiple NHANES cycles, using consistent food-code definitions, are warranted in future studies to better characterize changes in avocado consumption over time.

Although avocado is considered an episodically consumed fruit, the proportion of individuals who report consuming any avocado on a given day is relatively comparable to that of other commonly consumed fruits. In NHANES 2017–2018, on any given day, 12% of US adults aged ≥19 years reported consuming bananas, followed by apples (8%), grapes (6%), oranges (6%), strawberries (4%), and mixed fruit (4%) [27]. Among children and adolescents, apples were the most commonly consumed fruit (19%), followed by bananas (10%), oranges (9%), grapes (7%), strawberries (7%), and watermelon (4%) [28]. It is also important to compare avocado consumption with vegetables, given that the USDA’s MyPlate program classifies avocados as vegetables. In the same survey cycle, 26% of adults reported consuming potatoes—primarily in the form of fries—followed by salad (18%), tomatoes (5%), carrots (5%), broccoli (4%), corn (4%), string beans (4%), and mixed vegetables (4%) [29]. Similarly, among children and adolescents, potatoes (mostly fries) were the most commonly consumed vegetable (27%), followed by salad (7%), broccoli (5%), carrots (5%), and corn (4%) [30].

The demographic patterns observed among individuals who reported consuming any avocado on a given day are consistent with findings from earlier NHANES cycles and consumer tracking reports [6,12,31,32,33]. The likelihood of reporting any avocado consumption was higher among women, individuals who were married, and those with higher educational attainment and income levels. This trend was also observed in studies of general fruit and vegetable consumption [27,28,29,30,34,35]. These differences likely reflect broader dietary behaviors and sociodemographic factors. Individuals with higher education and income may have greater access to a wider variety of foods, increased exposure to nutrition information, and more opportunities for home food preparation, all of which can influence food choice and consumption patterns [36,37,38]. Similarly, differences by sex may be associated with variations in dietary preferences, household food purchasing practices, and meal preparation behaviors [39,40]. Racial and ethnic differences were also evident and in line with previous NHANES-based studies on avocado [6,12]. Most avocado consumers identified as non-Hispanic White and Hispanic. Additionally, the findings show that avocado consumption rates were highest among Hispanic, whereas non-Hispanic Black individuals exhibited the lowest consumption. The higher prevalence observed among Hispanic populations may reflect longstanding cultural dietary practices in which avocados are commonly incorporated into traditional cuisines [41,42]. These patterns are also consistent with broader fruit intake trends, which show the highest intake among non-Hispanic Asians and Hispanics, followed by non-Hispanic Whites and non-Hispanic Blacks [27,28]. However, the pattern differs for vegetables, where intake was highest among non-Hispanic Asians and Whites, followed by non-Hispanic Blacks and Hispanics [29,30]. From a public health perspective, these findings suggest that longstanding dietary practices among Hispanic populations, in which avocados are commonly consumed, may be important to consider when developing or evaluating nutrition strategies, while also highlighting the need to better understand barriers to avocado and fruit intake among populations with lower consumption [43].

Although the mean avocado intake observed in this analysis amounted to slightly less than half of a medium avocado (75 g), it contributed meaningfully to daily nutrient intake. Among avocado users, on average, avocado consumption accounted for more than 10% of the mean daily intake of several key nutrients, including beta-cryptoxanthin (33.4 ± 1.5%), alpha-carotene (27.0 ± 1.3%), dietary fiber (19.9 ± 0.6%), monounsaturated fatty acids (19.7 ± 0.5%), lutein + zeaxanthin (16.8 ± 0.7), vitamin K (14.1 ± 0.6%), vitamin E (alpha-tocopherol) (13.8 ± 0.5%), vitamin C (12.9 ± 0.7%), folate, expressed as dietary folate equivalents (12.5 ± 0.4%), potassium (11.4 ± 0.3%), and copper (10.1 ± 0.3%). These contributions are particularly relevant given that a large proportion of the US population fails to meet recommended intake levels for some of these nutrients. According to NHANES 2017–March 2020 data (usual intake from food and beverages), 44% of individuals aged one years and older did not meet the Estimated Average Requirement (EAR) for vitamin C and 74% fell short for vitamin E [25]. Moreover, only 28% exceeded the Adequate Intake (AI) for potassium, 6% for dietary fiber, and 54% for vitamin K [25]. Both potassium and dietary fiber are classified as nutrients of public health concern in the 2020–2025 Dietary Guidelines for Americans [44]. Notably, 67 g of avocado provides 339.7 mg of potassium (7% daily value—DV), 4.6 g of dietary fiber (16% DV), 14.0 mcg of vitamin K (12% DV), 5.9 mg of vitamin C (7% DV), and 1.3 mg of vitamin E (9% DV) [1].

These nutrient contributions illustrate how avocados contribute to dietary recommendations, particularly within underconsumed nutrient categories. Consistent with these findings, previous research has shown that avocado consumers tend to have higher intakes of potassium, dietary fiber, vitamins K, C, and E, compared to non-consumers [13]. However, interpretation of these findings should be contextualized relative to other fruits and vegetables. Most prior studies examining individual fruits or vegetables have focused on differences in total energy intake, overall nutrient intake, or nutrient adequacy among consumers versus non-consumers [45,46,47,48], rather than quantifying the relative contribution of a specific food to daily energy and nutrient intakes, as was done in the present analysis. Therefore, future research should apply similar contribution-based approaches to other individual fruits and vegetables to help provide a more comprehensive basis for comparison. To provide context in the interim, Table A2 in the Appendix A presents nutrient composition per 100 g of avocados relative to the top five commonly consumed individual fruits and vegetables determined by Hoy et al. using NHANES 2017–2018 [27,28,29,30]. Compared with most fruits and vegetables, avocados are more energy-dense and provide higher amounts of total fat—predominantly monounsaturated fatty acids—as well as relatively higher dietary fiber, magnesium, potassium, folate, niacin, and vitamin E per 100 g. Although avocados are relatively energy-dense, existing randomized controlled trials and observational studies do not indicate an association between avocado consumption and weight gain [49,50,51,52].

Interpretation of avocado nutrient contributions should also consider broader contextual factors, including affordability, accessibility, and sustainability. When compared with the top five commonly consumed individual fruits and vegetables, the average price per cup equivalent in 2023 was among the highest for corn, broccoli, strawberry, and avocado (Table A3). In terms of accessibility, increased imports have contributed to year-round availability of avocados in the U.S. food supply [11]. From a sustainability perspective, avocados are tree-grown crops, and their production may incorporate agricultural practices that support natural resource management, including soil and ecosystem stewardship, depending on growing region and management practices [53].

In addition to their nutrient contributions, insights into how and when avocados are consumed provide important context for their role in the diet. In the present analysis, dinner was the most common eating occasion for avocado consumption, followed by lunch, breakfast, and snacks. This pattern aligns with findings from a consumer tracking report [33] and NHANES data on vegetable consumption among children, adolescents, and adults [29,30], which also identified dinner and lunch as the most frequent occasions, followed by snacks and breakfast. In contrast, fruit consumption among adults typically occurred during snacks, then breakfast, lunch, and dinner [27]. Similarly, a separate NHANES analysis reported that children and adolescents most often consumed fruits as snacks, followed by lunch, breakfast, and dinner [28]. Most avocado consumption in the present study took place at home. Supporting this, the consumer tracking report found that over 60% of participants purchased avocados to eat or serve at home at least a few times per month [33]. Avocados were primarily sourced from supermarkets and convenience stores, consistent with retail data indicating that 60–65% of sales occur through these outlets [54]. These findings suggest that retail availability/accessibility and home preparation play significant roles in consumption patterns. Regarding preparation, plain avocado was the most reported form, followed by guacamole, avocado in sandwiches, and guacamole with tomatoes. This differs slightly from consumer tracking data, which identified guacamole/dips as the most frequent preparation, followed by use in salads, sandwiches/wraps/burgers, and plain avocado [33]. One possible explanation for this discrepancy may lie in differences in the sampled populations. NHANES uses a complex, probability-based sampling design to ensure representation of the entire U.S. population across all age groups [16,17,18]. In contrast, the consumer tracking data were limited to individuals aged 18 years or older who shared grocery shopping responsibilities and were already aware of avocados [33]. The inclusion criteria in the latter study may have selected for more engaged or health-conscious consumers, potentially influencing reported preparation preferences.

The study received funding from the Avocado Nutrition Center (ANC), and some authors are affiliated with the organization as employees or advisors. To ensure transparency and minimize potential bias, the study protocol and analytic plan were developed and preregistered prior to data analysis on the Open Science Framework [23], and all analyses were conducted using publicly available NHANES data collected independently by the National Center for Health Statistics [16]. Although the ANC supports research related to Hass avocados, NHANES dietary data do not distinguish between avocado varieties; therefore, the analyses reflect consumption of all avocado types reported by participants. In addition, the study followed established reporting guidelines (STROBE) [24], and interpretation of findings was limited to descriptive characterization of avocado consumption patterns and nutrient contributions, without extrapolation to dietary recommendations or health outcomes.

The present study has several strengths. It draws on data from a large, nationally representative health and nutrition survey, which enhances the generalizability of the results. Moreover, dietary intake was assessed using the USDA’s Automated Multiple-Pass Method—a validated technique designed to reduce bias and improve recall accuracy [55]. Nonetheless, several limitations should be considered. First, reliance on 24-h dietary recall introduces the possibility of recall bias, as individuals may forget or omit certain food items [56]. Second, the relatively small sample of avocado consumers in the non-Hispanic Black and other race groups may limit the reliability of the comparison of results to these groups. Lastly, the analysis was limited to the two most recent NHANES cycles to reflect current consumption patterns, which may restrict the ability to evaluate trends or behaviors preceding this period. Future studies incorporating a broader range of survey years are warranted to examine trends in avocado consumption over time. Lastly, analyses were restricted to Day 1 dietary recall data and avocado consumers only, which precludes estimation of usual intake distributions or assessment of population-level nutrient adequacy [16]. This analytic approach was intentionally aligned with the study objective of characterizing avocado consumption on a given day, including prevalence of consumption, amounts consumed, forms of intake, eating occasions, and associated nutrient contributions. Accordingly, the findings should be interpreted as descriptive of consumption days rather than indicative of the potential effects of increasing avocado intake at the population level.

## 5. Conclusions

In this nationally representative data, fewer than 6% of individuals in the US reported consuming avocado on a given day. However, avocado consumption was higher among certain demographic groups, including women, adults aged 19–50 years, and Hispanic populations. Among consumers, avocado intake averaged 67 g (or slightly less than half of a medium avocado) per day, with plain avocado as the most common form and dinner as the most frequent eating occasion in which avocado was consumed. Although the overall prevalence of consumption was modest, avocados contributed substantially to the daily intake of several underconsumed nutrients for consumers, such as dietary fiber, and vitamins C and E. These findings characterize current avocado consumption patterns and nutrient contributions among U.S. consumers but do not evaluate overall diet quality or health outcomes. Further research is needed to assess how avocado consumption fits within broader dietary patterns and whether changes in intake are associated with improvements in nutrient adequacy at the population level.

## Figures and Tables

**Table 1:** Percentages of participants consuming avocado by demographic characteristics (**A**) and total daily energy and macronutrient intake by avocado consumption status (**B**): National Health and Nutrition Examination Survey (NHANES) 2017-March 2020 Pre-pandemic and August 2021–August 2023 (n = 19,086).

**A. Percentages of Participants Consuming Avocado**					
**Demographic Characteristics**			**Total Population**	**Avocado Consumers**	***p*** **-Value**
			**n**	**n**	**% ± SE ^1^**
Overall		19,086	965	5.8 ± 0.3	
Age (yr) (%)					<0.0001
<19		6303	182	3.1 ± 0.3	
19–50		5748	402	7.4 ± 0.5	
>50		7035	381	5.5 ± 0.6	
Sex (%)					<0.0001
Men		9184	367	4.5 ± 0.3	
Women		9902	598	6.9 ± 0.5	
Race/ethnicity (%)					<0.0001
Hispanic		4081	351	9.1 ± 0.6	
Non-Hispanic White		8059	404	5.4 ± 0.5	
Non-Hispanic Black		4154	75	2.8 ± 0.5	
Other Race—Including Multi-Racial		2792	135	5.4 ± 0.7	
Marital Status (%) ^2^					0.1149
Married/Living with partner		7067	483	7.0 ± 0.5	
Widowed/Divorced/Separated		2990	148	5.3 ± 0.7	
Never married		2439	135	6.3 ± 0.8	
Education (%) ^2^					<0.0001
Less than 9th grade or 9–11th grade		1887	100	5.0 ± 0.7	
High school grad/GED or equivalent		2826	92	3.5 ± 0.6	
Some college or AA degree or College graduate or above		7783	575	8.0 ± 0.5	
Family income to poverty ratio (%)					<0.0001
<131%		5252	161	3.7 ± 0.5	
131–350%		6232	292	4.9 ± 0.5	
>350%		4089	327	8.0 ± 0.8	
**B. Total Daily Energy and Macronutrient Intake**					
		**Mean ± Standard Error**			
**Dietary Intake**		**Total Population** **(n = 19,076)**	**Avocado Non-Consumers** **(n = 18,111)**	**Avocado Consumers** **(n = 965)**	***p*** **-Value**
Energy	kcal	2039.3 ± 9.0	2031.3 ± 9.5	2169.5 ± 42.6	0.0041
Total fat	g	84.0 ± 0.5	83.4 ± 0.5	93.8 ± 1.7	<0.0001
	% energy	36.5 ± 0.1	36.4 ± 0.1	38.9 ± 0.4	<0.0001
Total Carbohydrate	g	237.3 ± 1.2	237.0 ± 1.3	242.2 ± 6.7	0.4511
	% energy	47.1 ± 0.1	47.3 ± 0.1	44.8 ± 0.4	<0.0001
Dietary fiber	g	15.8 ± 0.2	15.3 ± 0.2	24.5 ± 1.0	<0.0001
	g/1000 kcal	8.0 ± 0.1	7.8 ± 0.1	11.6 ± 0.3	<0.0001
Total Protein	g	75.7 ± 0.5	75.4 ± 0.5	81.0 ± 1.8	0.0037
	% energy	15.1 ± 0.1	15.1 ± 0.1	15.2 ± 0.2	0.7592

^1^ SE, standard errors. ^2^ Participants 20 years old or older.

**Table 2:** Absolute and energy-adjusted (per 1000 kcal) mean daily intake of avocado and its contribution to energy and nutrient intakes among avocado consumers in the National Health and Nutrition Examination Survey (NHANES), 2017–March 2020 (Pre-Pandemic) and August 2021–August 2023 (n = 965) ^1^.

		**Mean ± Standard Error**									
		**Overall (n = 965)**	**Sex**					**Age Group (Years) ^2^**			
			**Male** **(n = 367)**		**Female** **(n = 598)**	***p*-Value** ^4^		**<19 (n = 182)**	**19** **–** **50 (n = 402)**		**>50 (n = 381)**
Total avocado	g	67 ± 2.9	75.1 ± 5.4		61.9 ± 2.0	N/A		62.9 ± 3.7	66.7 ± 5.1		69.0 ± 3.2
	g/1000 kcal	33.6 ± 1.0	32.3 ± 1.9		34.4 ± 1.0	0.2955		36.4 ± 2.2	30.8 ± 1.8		36.9 ±1.7
Energy	kcal	105.3 ± 4.4	117.9 ± 8.4		97.4 ± 3.1	N/A		98.7 ± 5.8	104.8 ± 8		108.5 ± 4.9
	kcal/1000 kcal	52.9 ± 1.6	50.8 ± 3.0		54.1 ± 1.6	0.2947		57.1 ± 3.6	48.5 ±2.8		58.2 ± 2.6
Total fat	g	9.6 ± 0.4	10.8 ± 0.8		8.9 ± 0.3	N/A		9 ± 0.5	9.6 ± 0.7		9.9 ± 0.4
	g/1000 kcal	4.8 ± 0.1	4.6 ± 0.3		5.0 ± 0.1	0.2942		5.2 ± 0.3	4.4 ± 0.3		5.3 ± 0.2
Saturated fat	g	1.4 ± 0.1	1.6 ± 0.1		1.3 ± 0.0	N/A		1.3 ± 0.1	1.4 ± 0.1		1.4 ± 0.1
	g/1000 kcal	0.7 ± 0.0	0.7 ± 0.0		0.7 ± 0.0	0.2949		0.8 ± 0.0	0.6 ± 0.0		0.8 ± 0.0
Polyunsaturated fat	g	1.2 ± 0.0	1.3 ± 0.1		1.1 ± 0.0	N/A		1.1 ± 0.1	1.2 ± 0.1		1.2 ± 0.1
	g/1000 kcal	0.6 ± 0.0	0.6 ± 0.0		0.6 ± 0.0	0.2946		0.6 ± 0.0	0.5 ± 0.0		0.7 ± 0.0
Monounsaturated fat	g	6.4 ± 0.3	7.2 ± 0.5		5.9 ± 0.2	N/A		6 ± 0.4	6.4 ± 0.5		6.6 ± 0.3
	g/1000 kcal	3.2 ± 0.1	3.1 ± 0.2		3.3 ± 0.1	0.2952		3.5 ± 0.2	3.0 ± 0.2		3.6 ±0.2
Total Carbohydrate	g	5.7 ± 0.2	6.3 ± 0.5		5.2 ± 0.2	N/A		5.3 ± 0.3	5.6 ± 0.4		5.8 ± 0.3
	g/1000 kcal	2.8 ± 0.1	2.7 ± 0.2		2.9 ± 0.1	0.2987		3.1 ± 0.2	2.6 ± 0.2		3.1 ± 0.1
Dietary fiber	g	4.4 ± 0.2	4.9 ± 0.4		4.1 ± 0.1	N/A		4.1 ± 0.2	4.4 ± 0.3		4.5 ± 0.2
	g/1000 kcal	2.2 ± 0.1	2.1 ± 0.1		2.3 ± 0.1	0.2926		2.4 ± 0.1	2.0 ± 0.1		2.4 ± 0.1
Total Protein	g	1.3 ± 0.1	1.5 ± 0.1		1.2 ± 0.0	N/A		1.2 ± 0.1	1.3 ± 0.1		1.4 ± 0.1
	g/1000 kcal	0.7 ± 0.0	0.6 ± 0.0		0.7 ± 0.0	0.2911		0.7 ± 0.0	0.6 ± 0.0		0.7 ± 0.0
Copper	mg	0.1 ± 0.0	0.1 ± 0.0		0.1 ± 0.0	N/A		0.1 ± 0	0.1 ± 0		0.1 ± 0
	mg/1000 kcal	0.1 ± 0.0	0.1 ±0.0		0.1 ± 0.0	0.2871		0.1 ± 0.0	0.1 ± 0.0		0.1 ± 0.0
Magnesium	mg	19.2 ± 0.8	21.5 ± 1.5		17.7 ± 0.6	N/A		18 ± 1.1	19.1 ± 1.5		19.8 ± 0.9
	mg/1000 kcal	9.6 ± 0.3	9.3 ± 0.5		9.9 ± 0.3	0.3072		10.4 ± 0.7	8.8 ± 0.5		10.6 ± 0.5
Potassium	mg	320.4 ± 13.5	358.8 ± 25.4		296.4 ± 9.6	N/A		300.5 ± 17.8	319 ± 24.4		330.4 ± 14.9
	mg/1000 kcal	160.9 ± 4.9	154.7 ± 9.0		164.8 ± 4.7	0.2897		174.0 ± 10.8	147.5 ± 8.7		177.1 ± 8.1
Carotene, beta	µg	43.5 ± 1.9	48.5 ± 3.4		40.3 ± 1.4	N/A		41.2 ± 2.5	43.1 ± 3.3		44.8 ± 2.3
	µg/1000 kcal	21.7 ± 0.7	20.8 ± 1.2		22.3 ± 0.7	0.2158		23.8 ± 1.5 ^a^	19.8 ± 1.2 ^b^		23.9 ± 1.2 ^ab^
Carotene, alpha	µg	16.3 ± 0.7	18.3 ± 1.3		15.1 ± 0.5	N/A		15.5 ± 0.9	16.2 ± 1.2		16.9 ± 0.8
	µg/1000 kcal	8.2 ± 0.3	7.8 ± 0.5		8.4 ± 0.2	0.2297		8.9 ± 0.5	7.5 ± 0.4		9.0 ± 0.4
Cryptoxanthin, beta	µg	18.4 ± 0.8	20.6 ± 1.5		17.0 ± 0.5	N/A		17.2 ± 1.0	18.3 ± 1.4		19.0 ± 0.8
	µg/1000 kcal	9.2 ± 0.3	8.9 ± 0.5		9.5 ± 0.3	0.2929		10.0 ± 6.0	8.5 ± 0.5		10.2 ± 0.5
Lutein + zeaxanthin	µg	178.6 ± 7.5	199.9 ± 14.2		165.2 ± 5.3	N/A		167.4 ± 9.9	177.7 ± 13.6		184.2 ± 8.3
	µg/1000 kcal	89.7 ± 2.7	86.2 ± 5.0		91.8 ± 2.6	0.2886		96.9 ± 6.0	82.2 ± 4.8		98.8 ± 4.5
Folate, DFE ^3^	mcg	53.3 ± 2.2	59.7 ± 4.3		49.3 ± 1.6	N/A		49.9 ± 3	53.1 ± 4.1		55 ± 2.5
	mcg/1000 kcal	26.8 ± 0.8	25.8 ± 1.5		27.4 ± 0.8	0.2972		28.9 ± 1.8	24.6 ± 1.4		29.5 ± 1.3
Niacin	mg	1.1 ± 0.0	1.3 ± 0.1		1.1 ± 0.0	N/A		1.1 ± 0.1	1.1 ± 0.1		1.2 ± 0.1
	mg/1000 kcal	0.6 ± 0.0	0.6 ± 0.0		0.6 ± 0.0	0.2904		0.6 ± 0.0	0.5 ± 0.0		0.6 ± 0.0
Vitamin B-6	mg	0.2 ± 0.0	0.2 ± 0.0		0.2 ± 0.0	N/A		0.2 ± 0	0.2 ± 0		0.2 ± 0
	mg/1000 kcal	0.1 ± 0.0	0.1 ± 0.0		0.1 ± 0.0	0.2945		0.1 ± 0.0	0.1 ± 0.0		0.1 ± 0.0
Vitamin C	mg	6.8 ± 0.3	7.6 ± 0.5		6.3 ± 0.2	N/A		6.4 ± 0.4	6.7 ± 0.5		7 ± 0.3
	mg/1000 kcal	3.4 ± 0.1	3.3 ± 0.2		3.5 ± 0.1	0.2954		3.7 ± 0.2	3.1 ± 0.2		3.7 ± 0.2
Vitamin E	mg	1.4 ± 0.1	1.5 ± 0.1		1.3 ± 0.0	N/A		1.3 ± 0.1	1.4 ± 0.1		1.4 ± 0.1
	mg/1000 kcal	0.7 ± 0.0	0.7 ± 0.0		0.7 ± 0.0	0.2927		0.7 ± 0.0	0.6 ± 0.0		0.8 ± 0.0
Vitamin K	µg	13.8 ± 0.6	15.5 ± 1.1		12.8 ± 0.4	N/A		13.0 ± 0.8	13.8 ± 1.0		14.3 ± 0.6
	µg/1000 kcal	7.0 ± 0.2	6.7 ± 0.4		7.1 ± 0.2	0.2858		7.5 ± 0.5	6.4 ± 0.4		7.7 ± 0.3
		**Mean ± standard error**									
		**Race/ethnicity ^2^**									
		**Mexican American or Other Hispanic (n = 351)**		**Non-Hispanic White (n = 404)**			**Non-Hispanic Black (n = 75)**			**Other Race—Including Multi-Racial (n = 135)**	
Total avocado	g	66.5 ± 3.4		63.5 ± 4.4			74.8 ± 7.4			82.5 ± 7.9	
	g/1000 kcal	32.9 ± 1.5		31.3 ± 1.4			40.9 ± 3.3			43.6 ± 4.6	
Energy	kcal	104.8 ± 5.5		99.5 ± 6.7			117.2 ± 11.2			130.4 ± 12.5	
	kcal/1000 kcal	51.9 ± 2.4		49.2 ± 2.2			64.4 ± 5.2			68.9 ± 7.3	
Total fat	g	9.6 ± 0.5		9.1 ± 0.6			10.7 ± 1.0			11.9 ± 1.1	
	g/1000 kcal	4.7 ± 0.2		4.5 ± 0.2			5.9 ± 0.5			6.3 ± 0.7	
Saturated fat	g	1.4 ± 0.1		1.3 ± 0.1			1.6 ± 0.1			1.7 ± 0.2	
	g/1000 kcal	0.7 ± 0.0		0.7 ± 0.0			0.9 ± 0.1			0.9 ± 0.1	
Polyunsaturated fat	g	1.2 ± 0.1		1.1 ± 0.1			1.3 ± 0.1			1.5 ± 0.1	
	g/1000 kcal	0.6 ± 0.0		0.6 ± 0.0			0.7 ± 0.1			0.8 ± 0.1	
Monounsaturated fat	g	6.4 ± 0.3		6.1 ± 0.4			7.2 ± 0.7			8.0 ± 0.8	
	g/1000 kcal	3.2 ± 0.1		3.0 ± 0.1			3.9 ± 0.3			4.2 ± 0.4	
Total Carbohydrate	g	5.6 ± 0.3		5.4 ± 0.4			6.3 ± 0.6			7.0 ± 0.7	
	g/1000 kcal	2.8 ± 0.1		2.6 ± 0.1			3.5 ± 0.3			3.7 ± 0.4	
Dietary fiber	g	4.4 ± 0.2		4.2 ± 0.3			4.9 ± 0.5			5.5 ± 0.5	
	g/1000 kcal	2.2 ± 0.1		2.1 ± 0.1			2.7 ± 0.2			2.9 ± 0.3	
Total Protein	g	1.3 ± 0.1		1.3 ± 0.1			1.5 ± 0.1			1.6 ± 0.2	
	g/1000 kcal	0.7 ± 0.0		0.6 ± 0.0			0.8 ± 0.1			0.9 ± 0.1	
Copper	mg	0.1 ± 0.0		0.1 ± 0.0			0.1 ± 0.0			0.2 ± 0.0	
	mg/1000 kcal	0.1 ± 0.0		0.1 ± 0.0			0.1 ± 0.0			0.1 ± 0.0	
Magnesium	mg	19.1 ± 1.0		18.1 ± 1.2			21.4 ± 2.1			23.9 ± 2.3	
	mg/1000 kcal	9.4 ± 0.4		9.0 ± 0.4			11.7 ± 1.0			12.6 ± 1.3	
Potassium	mg	319.0 ± 16.8		303.1 ± 20.5			357.5 ± 34.5			396.7 ± 38.0	
	mg/1000 kcal	157.8 ± 7.3		149.6 ± 6.8			196.2 ± 15.8			209.6 ± 22.3	
Carotene, beta	µg	43.1 ± 2.1		41.1 ± 2.9			49.8 ± 5.8			53.7 ± 5.1	
	µg/1000 kcal	21.3 ± 0.9		20.2 ± 1.0			26.8 ± 2.4			28.2 ± 3.0	
Carotene, alpha	µg	16.2 ± 0.8		15.4 ± 1.1			18.5 ± 2.0			20.2± 1.9	
	µg/1000 kcal	8.0 ± 0.4		7.6 ± 0.4			10.0 ± 0.8			10.6 ± 1.1	
Cryptoxanthin, beta	µg	18.3 ± 1.0		17.4 ± 1.2			20.4 ± 2.0			22.8 ± 2.2	
	µg/1000 kcal	9.1 ± 0.4		8.6 ± 0.4			11.2 ± 0.9			12.0 ± 1.3	
Lutein + zeaxanthin	µg	177.9 ± 9.4		168.8 ± 11.4			199.2 ± 19.2			221.2 ± 21.3	
	µg/1000 kcal	88.0 ± 4.1		83.4 ± 3.8			109.4 ± 8.8			116.9 ± 12.4	
Folate, DFE ^3^	µg	53.1 ± 2.8		50.4 ± 3.4			59.5 ± 5.7			66.1 ± 6.4	
	µg/1000 kcal	26.3 ± 1.2		24.9 ± 1.1			32.6 ± 2.6			35.0 ± 3.7	
Niacin	mg	1.1 ± 0.1		1.1 ± 0.1			1.3 ± 0.1			1.4 ± 0.1	
	mg/1000 kcal	0.6 ± 0.0		0.5 ± 0.0			0.7 ± 0.1			0.7 ± 0.1	
Vitamin B-6	mg	0.2 ± 0.0		0.2 ± 0.0			0.2 ± 0.0			0.2 ± 0.0	
	mg/1000 kcal	0.1 ± 0.0		0.1 ± 0.0			0.1 ± 0.0			0.1 ± 0.0	
Vitamin C	mg	6.7 ± 0.3		6.4 ± 0.5			7.6 ± 0.8			8.3 ± 0.8	
	mg/1000 kcal	3.3 ± 0.1		3.2 ± 0.1			4.1 ± 0.3			4.4 ± 0.5	
Vitamin E	mg	1.4 ± 0.1		1.3 ± 0.1			1.5 ± 0.1			1.7 ± 0.2	
	mg/1000 kcal	0.7 ± 0.0		0.6 ± 0.0			0.8 ± 0.1			0.9 ± 0.1	
Vitamin K	µg	13.8 ± 0.8		13.1 ± 0.9			15.4 ± 1.5			17.1 ± 1.6	
	µg/1000 kcal	6.8 ± 0.3		6.5 ± 0.3			8.5 ± 0.7			9.1 ± 1.0	

^1^ Table displays only macronutrients and selected nutrients contributing ≥5% of total nutrient intake. Absolute intake values are presented descriptively. *p*-values are shown only for energy-adjusted intake (per 1000 kcal), which accounts for differences in total energy intake across groups. ^2^ Different superscript letters (e.g., a, b) indicate statistically significant differences between groups (*p* < 0.05) based on the Tukey–Kramer post hoc comparisons. ^3^ DFE, Dietary Folate Equivalents. ^4^ N/A, not applicable.

**Table 3:** Percentages of mean daily energy and nutrient intakes from avocado among avocado consumers in the National Health and Nutrition Examination Survey (NHANES), 2017–March 2020 (Pre-Pandemic) and August 2021–August 2023 (n = 965) ^1^.

	**Percentage of Mean Intake ± Standard Error**									
	**Overall (n = 965)**	**Sex**					**Age Group ^2^**			
		**Male (n = 367)**		**Female (n = 598)**	***p*** **-Value**		**<19 (n = 182)**	**19–50 (n = 402)**		**>50 (n = 381)**
Energy	5.3 ± 0.2	5.1 ± 0.3		5.4 ± 0.2	0.2947		5.7 ± 0.4	4.8 ± 0.3		5.8 ± 0.3
Total fat	11.4 ± 0.4	11.2 ± 0.7		11.5 ± 0.4	0.6222		11.9 ± 0.7	10.4 ± 0.6		12.8 ± 0.6
Saturated fat	6.3 ± 0.3	6.2 ± 0.5		6.3 ± 0.3	0.7784		5.9 ± 0.4 ^a^	5.6 ± 0.4 ^a^		7.5 ± 0.6 ^b^
Polyunsaturated fat	7.1 ± 0.3	7.0 ± 0.5		7.1 ± 0.3	0.8333		8.0 ± 0.6	6.4 ± 0.5		7.9 ± 0.5
Monounsaturated fat	19.7 ± 0.5	19.3 ± 0.9		19.9 ± 0.6	0.5700		21.1 ± 1.2	18.2 ± 0.9		21.6 ± 0.9
Total Carbohydrate	2.8 ± 0.1	2.7 ± 0.2		2.9 ± 0.1	0.4687		2.7 ± 0.2	2.7 ± 0.2		3.0 ± 0.1
Dietary fiber	19.9 ± 0.6	20.2 ± 0.8		19.7 ± 0.7	0.5474		22.0 ± 1.0	19.3 ± 0.9		20.1 ± 0.7
Total Protein	1.9 ± 0.1	1.8 ± 0.1		2.0 ± 0.1	0.2555		2.1 ± 0.1	1.7 ± 0.1		2.1 ± 0.1
Copper	10.1 ± 0.3	10.3 ± 0.5		9.9 ± 0.4	0.3445		12.1 ± 0.7 ^a^	9.6 ± 0.6 ^b^		10.1 ± 0.4 ^b^
Magnesium	6.1 ± 0.2	6.1 ± 0.3		6.1 ± 0.2	0.9212		7.3 ± 0.4 ^a^	5.7 ± 0.4 ^b^		6.2 ± 0.3 ^ab^
Potassium	11.4 ± 0.3	11.5 ± 0.5		11.3 ± 0.4	0.8247		13.1 ± 0.7	11.0 ± 0.6		11.3 ± 0.4
Carotene, beta	6.2 ± 0.4	7.3 ± 0.8		5.5 ± 0.4	0.0774		9.2 ± 1.0 ^a^	5.8 ± 0.5 ^b^		5.8 ± 0.9 ^b^
Carotene, alpha	27.0 ± 1.3	29.4 ± 1.9		25.6 ± 1.6	0.1097		30.6 ± 2.7	26.9 ± 1.8		25.9 ± 2.1
Cryptoxanthin, beta	33.4 ± 1.5	35.3 ± 2.3		32.2 ± 1.9	0.2772		35.0 ± 2.6	34.6 ± 2.1		30.8 ± 2.0
Lutein + zeaxanthin	16.8 ± 0.7	19.0 ± 1.1		15.3 ± 0.9	0.0136		21.6 ± 1.4 ^a^	16.4 ± 1.1 ^b^		15.6 ± 1.3 ^b^
Folate, DFE ^3^	12.5 ± 0.4	12.8 ± 0.8		12.3 ± 0.5	0.5368		13.3 ± 0.8	12.1 ± 0.7		12.7 ± 0.6
Niacin	5.6 ± 0.2	5.2 ± 0.3		5.8 ± 0.2	0.0607		6.7 ± 0.5 ^a^	4.9 ± 0.3 ^b^		6.1 ± 0.3 ^ab^
Vitamin B-6	9.3 ± 0.3	8.8 ± 0.5		9.6 ± 0.4	0.1211		10.6 ± 0.5 ^a^	8.4 ± 0.6 ^b^		10.2 ± 0.5 ^ab^
Vitamin C	12.9 ± 0.7	14.6 ± 1.0		11.8 ± 0.9	0.0368		12.9 ± 1.3	13.2 ± 1.1		12.3 ±1.2
Vitamin E (alpha-tocopherol)	13.8 ± 0.5	14.4 ± 0.8		13.4 ± 0.5	0.2501		15.7 ± 0.9 ^a^	12.9 ± 0.8 ^b^		14.4 ± 0.7 ^ab^
Vitamin K	14.1 ± 0.6	15.2 ± 0.9		13.4 ± 0.7	0.0855		18.8 ± 1.0 ^a^	13.4 ± 0.9 ^b^		13.4 ± 0.9 ^b^
	**Percentage of mean intake ± standard errors**									
	**Race/ethnicity ^2^**									
	**Mexican American or Other Hispanic (n = 351)**		**Non-Hispanic White (n = 404)**			**Non-Hispanic Black (n = 75)**			**Other Race—Including Multi-Racial (n = 135)**	
Energy	5.2 ± 0.2		4.9 ± 0.2			6.4 ± 0.5			6.9 ± 0.7	
Total fat	11.2 ± 0.5		10.5 ± 0.5			14.4 ± 1.4			15.0 ± 1.8	
Saturated fat	5.9 ± 0.3 ^a^		5.7 ± 0.3 ^a^			8.7 ± 1.0 ^b^			8.9 ± 1.3 ^ab^	
Polyunsaturated fat	7.4 ± 0.4		6.4 ± 0.4			8.8 ± 1.1			8.9 ± 1.1	
Monounsaturated fat	19.4 ± 0.8		18.6 ± 0.7			23.0 ± 2.0			24.8 ± 2.7	
Total Carbohydrate	2.8 ± 0.2		2.6 ± 0.1			3.5 ± 0.4			3.5 ± 0.4	
Dietary fiber	20.3 ± 0.9 ^a^		18.3 ± 0.9 ^a^			25.5 ± 1.8 ^b^			24.2 ± 2.8 ^ab^	
Total Protein	1.8 ± 0.1		1.8 ± 0.1			2.6 ± 0.3			2.5 ± 0.3	
Copper	10.6 ± 0.4 ^a^		8.9 ± 0.5 ^a^			13.6 ± 1.1 ^b^			12.7 ± 1.6 ^ab^	
Magnesium	6.1 ± 0.3		5.6 ± 0.4			7.5 ± 0.7			7.9 ± 1.0	
Potassium	11.5 ± 0.4 ^ab^		10.4 ± 0.6 ^a^			14.4 ± 1.2 ^b^			14.3 ± 1.6 ^ab^	
Carotene, beta	6.6 ± 0.7		5.3 ± 0.6			8.5 ± 1.9			8.6 ± 1.6	
Carotene, alpha	27.6 ± 2.1		26.4 ± 1.7			30.5 ± 4.7			26.9± 3.6	
Cryptoxanthin, beta	33.7 ± 2.5		32.2 ± 1.8			38.9 ± 3.8			35.7 ± 4.6	
Lutein + zeaxanthin	18.2 ± 0.9		15.2 ± 1.2			20.4 ± 2.2			19.2 ± 2.9	
Folate, DFE ^3^	12.4 ± 0.6		11.6 ± 0.7			15.1 ± 1.7			15.7 ± 2.3	
Niacin	5.3 ± 0.3		5.2 ± 0.3			6.8 ± 0.7			7.4 ± 0.9	
Vitamin B-6	8.8 ± 0.4		8.8 ± 0.6			12.5 ± 1.5			11.7 ± 1.3	
Vitamin C	13.7 ± 1.1		12.0 ± 1.0			15.1 ± 2.2			13.8 ± 1.8	
Vitamin E (alpha-tocopherol)	14.7 ± 0.6		12.5 ± 0.7			15.2 ± 1.8			17.2 ± 1.9	
Vitamin K	16.0 ± 0.9		12.1 ± 0.9			18.0 ± 2.4			16.8 ± 2.3	

^1^ Table displays only macronutrients and selected nutrients contributing ≥5% of mean nutrient intake. Dietary reference intake values for selected key nutrients are provided here for contextual reference only. Percent contributions represent the proportion of daily intake derived from avocado among consumers on the recall day and should not be interpreted as indicators of usual intake or nutrient adequacy. Dietary fiber Adequate Intake (AI) = 25 g/day (women aged 19–50 years), 38 g/day (men aged 19–50 years); copper Estimated Average Requirement (EAR) = 0.7 mg/day (women and men aged +19 years); magnesium EAR = 265 mg/day (women aged 31–70 years), 350 mg/day (men aged 31–70 years); potassium AI = 2600 mg/day (women aged +19 years), 3400 mg/day (men aged +19 years); folate, DFE EAR = 320 µg/day (women and men aged +19 years); niacin EAR = 11 mg/day (women aged +19 years), 12 mg/day (men aged +19 years); vitamin B-6 EAR = 1.1 mg/day (women and men aged 19–50 years); vitamin C EAR = 60 mg/day (women aged +19 years), 75 mg/day (men aged +19 years); vitamin E (alpha-tocopherol) EAR = 12 mg/day (women and men aged +19 years); vitamin K AI = 90 µg/day (women aged +19 years) and 120 µg/day (men aged +19 years) [25]. ^2^ Different superscript letters (e.g., a, b) indicate statistically significant differences between groups (*p* < 0.05) based on the Tukey–Kramer post hoc comparisons. ^3^ DFE, Dietary Folate Equivalents.

## Data Availability

NHANES datasets are publicly available: http://www.cdc.gov/nchs/nhanes/ (accessed on 1 November 2024).

## Abbreviations

The following abbreviations are used in this manuscript:

USDA	United States Department of Agriculture
NHANES	National Health and Nutrition Examination Survey
NCHS	National Center for Health Statistics
CDC	Centers for Disease Control and Prevention
MEC	Mobile Examination Center
FNDDS	Food and Nutrient Database for Dietary Studies
EAR	Estimated Average Requirement
AI	Adequate Intake
DV	Daily Value
ANOVA	Analysis of Variance

## Appendix A

**Table A1:** Prevalence of avocado consumption under different food-code definitions in NHANES 2017–March 2020 and August 2021–August 2023.

Definition of Avocado Consumer	n	% ± SE ^1^
Avocado (avocado: 63105010 and avocado for use on a sandwich: 89902000—consistent with prior studies) ^2^	670	3.9 ± 0.3
Expanded definition (avocado: 63105010, guacamole: 63409010, avocado for use on a sandwich: 89902000, and guacamole with tomatoes: 63409015)	965	5.8 ± 0.3

^1^ SE, standard errors. ^2^ Prior to What We Eat in America/National Health and Nutrition Examination Survey and Food and Nutrient Database for Dietary Studies (WWEIA/NHANES and FNDDS) 2017–2018, avocado used on a sandwich was captured in the totals for food code 63105010 (avocado) because participants were asked what was on their sandwich. Beginning in WWEIA/NHANES and FNDDS 2017–2018, a separate food code (avocado on a sandwich: 89902000) was made to separate out these reports.

**Table A2:** Selected nutrient content per 100 g of avocado and the top five commonly consumed individual fruits and vegetables ^1^.

		Fruits					Vegetables				
	Avocado, Raw	Apple, Raw	Banana, Raw	Grape, Raw	Orange, Raw	Strawberry, Raw	Broccoli, Raw	Carrot, Raw	Corn, Fresh, Cooked, no Added Fat	Potato, Boiled, from Fresh, Peel not Eaten, no Added Fat	Tomato, Raw
FNDDS code ^2^	63105010	63101000	63107010	63123000	61119010	63223020	72201100	73101010	75216111	71103010	74101000
Energy (kcal)	160	61	97	83	50	36	39	44	86	93	20
Total fat (g)	14.66	0.15	0.28	0.2	0.14	0.22	0.34	0.24	1.35	0.1	0.31
Saturated fat (g)	2.126	0.028	0.112	0.054	0.015	0	0.039	0.028	0.324	0.026	0.028
Polyunsaturated fat (g)	1.816	0.051	0.073	0.048	0.025	0	0.017	0.083	0.486	0.043	0.083
Monounsaturated fat (g)	9.799	0.007	0.032	0.007	0.023	0	0.011	0.01	0.431	0.002	0.031
Total Carbohydrate (g)	8.53	14.8	22.71	19.4	11.78	7.96	6.27	9.68	18.64	21.5	4.04
Dietary fiber (g)	6.7	2.1	1.7	0.9	2.2	1.8	2.4	2.9	2	1.5	1.2
Total Protein (g)	2	0.17	0.74	0.9	0.92	0.64	2.57	0.87	3.26	1.95	0.82
Copper (mg)	0.19	0.03	0.101	0.071	0.054	0.035	0.059	0.051	0.054	0.214	0.051
Magnesium (mg)	29	5	28	8	10	13	21	12	37	25	10
Potassium (mg)	485	104	326	224	174	161	303	258	269	389	226
Carotene, beta (µg)	62	27	8	39	71	7	93	7338	45	0	397
Carotene, alpha (µg)	24	0	7	1	11	0	0	3622	15	0	61
Cryptoxanthin, beta (µg)	28	11	0	0	116	0	0	0	109	0	6
Lutein + zeaxanthin (µg)	271	29	22	72	129	22	745	307	610	0	103
Folate, DFE (mcg) ^2^	81	0	15	2	28	8	65	40	36	9	13
Niacin (mg)	1.738	0.091	0.653	0.188	0.354	0.386	0.639	1.074	1.676	1.39	0.597
Vitamin B-6 (mg)	0.257	0.03	0.212	0.086	0.07	0.035	0.191	0.13	0.083	0.3	0.078
Vitamin C (mg)	10	4.6	12	3.2	56.2	59.6	91.3	4.2	5.8	12.7	16.3
Vitamin E (alpha- tocopherol) (mg)	2.07	0.18	0.1	0.19	0.18	0.29	0.15	0.66	0.07	0.04	0.58
Vitamin K (µg)	21	2.2	0.1	14.6	0	2.1	102	11.3	0.3	0.3	7.5

^1^ The top five commonly consumed individual fruits and vegetables were identified according to Hoy et al. using NHANES 2017–2018 [27,28,29,30]; mixed dishes (e.g., salads) were excluded because of variability in composition. Nutrient values are from Food and Nutrient Database for Dietary Studies 2021–2023. ^2^ DFE, Dietary Folate Equivalents. FNDDS, Food and Nutrient Database for Dietary Studies.

**Table A3:** Average Price Per Cup Equivalent in 2023 of avocado and the top five commonly consumed individual fruits and vegetables ^1^.

	Average Price (USD) per Cup Equivalent ^2^
Avocado, fresh	0.976334492
Apple, fresh	0.501435057
Banana, fresh	0.31498274
Grape, fresh	0.77452409
Orange, fresh	0.863515806
Strawberry, fresh	1.046999868
Broccoli heads, fresh	1.324613857
Carrot, raw whole, fresh	0.311353821
Corn, fresh	1.416494534
Potato, fresh	0.300503543
Tomato, fresh	0.882356978

^1^ The top five commonly consumed individual fruits and vegetables were identified according to Hoy et al. [27,28,29,30]; mixed dishes (e.g., salads) were excluded because of variability in composition. ^2^ Fruit and vegetable price estimated by the U.S. Department of Agriculture Economic Research Service [57].

## References

[1] Dreher M.L., Davenport A.J. (2013). Hass Avocado Composition and Potential Health Effects. Crit. Rev. Food Sci. Nutr..

[2] Dreher M.L., Cheng F.W., Ford N.A. (2021). A Comprehensive Review of Hass Avocado Clinical Trials, Observational Studies, and Biological Mechanisms. Nutrients.

[3] Park E., Edirisinghe I., Burton-Freeman B. (2018). Avocado Fruit on Postprandial Markers of Cardio-Metabolic Risk: A Randomized Controlled Dose Response Trial in Overweight and Obese Men and Women. Nutrients.

[4] Wien M., Haddad E., Oda K., Sabaté J. (2013). A Randomized 3 × 3 Crossover Study to Evaluate the Effect of Hass Avocado Intake on Post-Ingestive Satiety, Glucose and Insulin Levels, and Subsequent Energy Intake in Overweight Adults. Nutr. J..

[5] Dreher M.L. (2018). Whole Fruits and Fruit Fiber Emerging Health Effects. Nutrients.

[6] Cheng F.W., Ford N.A., Taylor M.K. (2021). US Older Adults That Consume Avocado or Guacamole Have Better Cognition Than Non-Consumers: National Health and Nutrition Examination Survey 2011–2014. Front. Nutr..

[7] Pedreschi R., Uarrota V., Fuentealba C., Alvaro J.E., Olmedo P., Defilippi B.G., Meneses C., Campos-Vargas R. (2019). Primary Metabolism in Avocado Fruit. Front. Plant Sci..

[8] Daley S.F., Hinson M.R. (2025). Mediterranean Diet. StatPearls.

[9] Satija A., Hu F.B. (2018). Plant-Based Diets and Cardiovascular Health. Trends Cardiovasc. Med..

[10] Huang K.-M., Guan Z., Blare T., Hammami A.M. (2023). Global Avocado Boom. Choices.

[11] Imports Play Dominant Role as U.S. Demand for Avocados Climbs|Economic Research Service. https://ers.usda.gov/data-products/charts-of-note/chart-detail?chartId=103810.

[12] Fulgoni V., O’Neil C., Nicklas T. (2017). Avocado Consumption by Adults Is Associated with Better Nutrient Intake, Diet Quality, and Some Measures of Adiposity: National Health and Nutrition Examination Survey, 2001–2012. Intern. Med. Rev..

[13] Fulgoni V.L., Dreher M., Davenport A.J. (2013). Avocado Consumption Is Associated with Better Diet Quality and Nutrient Intake, and Lower Metabolic Syndrome Risk in US Adults: Results from the National Health and Nutrition Examination Survey (NHANES) 2001–2008. Nutr. J..

[14] Akinbam L., Chen T.-C., Davy O., Ogden C., Fink S., Clark J., Riddles M., Mohadjer L. (2022). National Health and Nutrition Examination Survey, 2017-March 2020 Prepandemic File: Sample Design, Estimation, and Analytic Guidelines.

[15] Moshfegh A.J., Rhodes D.G., Baer D.J., Murayi T., Clemens J.C., Rumpler W.V., Paul D.R., Sebastian R.S., Kuczynski K.J., Ingwersen L.A. (2008). The US Department of Agriculture Automated Multiple-Pass Method Reduces Bias in the Collection of Energy Intakes. Am. J. Clin. Nutr..

[16] Ahluwalia N., Dwyer J., Terry A., Moshfegh A., Johnson C. (2016). Update on NHANES Dietary Data: Focus on Collection, Release, Analytical Considerations, and Uses to Inform Public Policy. Adv. Nutr..

[17] Centers for Disease Control and Prevention (2017). National Health and Nutrition Examination Survey (NHANES): MEC In-Person Dietary Interviewers Procedures Manual.

[18] Zweig S. (2021). MEC ACASI Procedures Manual 2021.

[19] Food Surveys Research Group: USDA ARS. https://www.ars.usda.gov/northeast-area/beltsville-md-bhnrc/beltsville-human-nutrition-research-center/food-surveys-research-group/.

[20] NHANES Tutorials-NHANES Dietary Analyses Module. https://wwwn.cdc.gov/nchs/nhanes/tutorials/dietaryanalyses.aspx.

[21] NHANES August 2021–August 2023. https://wwwn.cdc.gov/nchs/nhanes/continuousnhanes/default.aspx?Cycle=2021-2023.

[22] NHANES 2017-March 2020 Pre-Pandemic. https://wwwn.cdc.gov/nchs/nhanes/continuousnhanes/default.aspx?Cycle=2017-2020.

[23] OSF|Avocado Consumption and Patterns Among Children and Adults: National Health and Nutrition Examination Survey 2017-March 2020 and August 2021–August 2023. https://osf.io/4vcbm/overview.

[24] Cuschieri S. (2019). The STROBE Guidelines. Saudi J. Anaesth..

[25] USDA, Agricultural Research Service (2023). Usual Nutrient Intake from Food and Beverages, by Males/Females and Age, What We Eat in America, NHANES 2017-March 2020 Prepandemic.

[26] Meals on Wheels. https://www.mealsonwheelsamerica.org/.

[27] Hoy M.K., Clemens J.C., Moshfegh A.J. (2010). Intake of Fruit by Adults: What We Eat in America, NHANES 2017–2018. FSRG Dietary Data Briefs.

[28] Hoy M.K., Clemens J.C., Moshfegh A.J. (2010). Intake of Fruit by Children and Adolescents: What We Eat in America, NHANES 2017–2018. FSRG Dietary Data Briefs.

[29] Hoy M.K., Clemens J.C., Moshfegh A.J. (2010). Intake of Vegetables by Adults: What We Eat in America, NHANES 2017–2018. FSRG Dietary Data Briefs.

[30] Hoy M.K., Clemens J.C., Moshfegh A.J. (2010). Intake of Vegetables by Children and Adolescents: What We Eat in America, NHANES 2017–2018. FSRG Dietary Data Briefs.

[31] Cheng F.W., Rodríguez-Ramírez S., Shamah-Levy T., Pérez-Tepayo S., Ford N.A. (2024). Association Between Avocado Consumption and Diabetes in Mexican Adults: Results from the 2012, 2016, and 2018 Mexican National Health and Nutrition Surveys. J. Acad. Nutr. Diet..

[32] Cheng F.W., Park S.-Y., Haiman C.A., Wilkens L.R., Le Marchand L., Ford N.A. (2024). Avocado and Guacamole Consumption and Colorectal Cancer Risk: The Multiethnic Cohort Study. Nutr. Cancer.

[33] Cooper Roberts Research (2023). Avocados Consumer Tracking 2023: Segment Report.

[34] Ansai N., Wambogo E.A. (2021). Fruit and Vegetable Consumption Among Adults in the United States, 2015–2018.

[35] Wambogo E.A., Ansai N., Ahulwalia N., Ogden C.L. (2020). Fruit and Vegetable Consumption Among Children and Adolescents in the United States, 2015–2018. NCHS Data Brief.

[36] Kirkpatrick S.I., Dodd K.W., Reedy J., Krebs-Smith S.M. (2012). Income and Race/Ethnicity Are Associated with Adherence to Food-Based Dietary Guidance among US Adults and Children. J. Acad. Nutr. Diet..

[37] Darmon N., Drewnowski A. (2008). Does Social Class Predict Diet Quality?. Am. J. Clin. Nutr..

[38] Hermosa-Bosano C., López-Gil J.F. (2025). Socioecological Correlates of Perceived Cooking Skills among Spanish Adolescents: The EHDLA Study. Front. Public Health.

[39] Wardle J., Haase A.M., Steptoe A., Nillapun M., Jonwutiwes K., Bellisle F. (2004). Gender Differences in Food Choice: The Contribution of Health Beliefs and Dieting. Ann. Behav. Med..

[40] Hiza H.A.B., Casavale K.O., Guenther P.M., Davis C.A. (2013). Diet Quality of Americans Differs by Age, Sex, Race/Ethnicity, Income, and Education Level. J. Acad. Nutr. Diet..

[41] Galindo-Tovar M.E., Arzate-Fernández A.M., Ogata-Aguilar N., Landero-Torres I. (2007). The Avocado (Persea Americana, Lauraceae) Crop in Mesoamerica: 10,000 Years of History. Harv. Pap. Bot..

[42] Dillehay T.D., Goodbred S., Pino M., Vásquez Sánchez V.F., Tham T.R., Adovasio J., Collins M.B., Netherly P.J., Hastorf C.A., Chiou K.L. (2017). Simple Technologies and Diverse Food Strategies of the Late Pleistocene and Early Holocene at Huaca Prieta, Coastal Peru. Sci. Adv..

[43] Jayasinghe S., Byrne N.M., Hills A.P. (2025). Cultural Influences on Dietary Choices. Progress Cardiovasc. Dis..

[44] Snetselaar L.G., de Jesus J.M., DeSilva D.M., Stoody E.E. (2021). Dietary Guidelines for Americans, 2020–2025. Nutr. Today.

[45] Fulgoni K., Fulgoni V.L. (2022). Watermelon Intake Is Associated with Increased Nutrient Intake and Higher Diet Quality in Adults and Children, NHANES 2003–2018. Nutrients.

[46] Nicklas T.A., O’Neil C.E., Fulgoni V.L. (2015). Consumption of Various Forms of Apples Is Associated with a Better Nutrient Intake and Improved Nutrient Adequacy in Diets of Children: National Health and Nutrition Examination Survey 2003–2010. Food Nutr. Res..

[47] Fulgoni K., Fulgoni V.L. (2024). Mango Consumption Was Associated with Higher Nutrient Intake and Diet Quality in Women of Childbearing Age and Older Adults. Nutrients.

[48] Agarwal S., Fulgoni V.L. (2021). Intake of Potatoes Is Associated with Higher Diet Quality, and Improved Nutrient Intake and Adequacy among US Adolescents: NHANES 2001–2018 Analysis. Nutrients.

[49] Clarke A.E., LeBeau K.S., Oda K., Segovia-Siapco G., Paalani M., Reboussin D.M., Lichtenstein A.H., Rajaram S., Sabaté J. (2024). The Effect of Daily Avocado Intake on Food and Nutrient Displacement in a Free-Living Population with Abdominal Obesity. Curr. Dev. Nutr..

[50] Segovia-Siapco G., Paalani M., Oda K., Pribis P., Sabaté J. (2021). Associations between Avocado Consumption and Diet Quality, Dietary Intake, Measures of Obesity and Body Composition in Adolescents: The Teen Food and Development Study. Nutrients.

[51] Conceição A.R., Fraiz G.M., Rocha D.M.U.P., Bressan J. (2022). Can Avocado Intake Improve Weight Loss in Adults with Excess Weight? A Systematic Review and Meta-Analysis of Randomized Controlled Trials. Nutr. Res..

[52] Luong T.Q., Adeyemo M.A., Kris-Etherton P.M., Lichtenstein A.H., Matthan N.R., Petersen K.S., Reboussin D.M., Sabaté J., Li Z. (2025). Adherence and Body Weight with Daily Avocado Consumption Among Latina Women of the Habitual Diet and Avocado Trial (HAT). Nutrients.

[53] The Avocado Sustainability Center. https://sustainability.hassavocadoboard.com/.

[54] Technomic (2019). US Avocado Volumetric Update: 2019.

[55] Raper N., Perloff B., Ingwersen L., Steinfeldt L., Anand J. (2004). An Overview of USDA’s Dietary Intake Data System. J. Food Compos. Anal..

[56] Gibson R.S., Charrondiere U.R., Bell W. (2017). Measurement Errors in Dietary Assessment Using Self-Reported 24-Hour Recalls in Low-Income Countries and Strategies for Their Prevention. Adv. Nutr..

[57] Fruit and Vegetable Prices|Economic Research Service. https://www.ers.usda.gov/data-products/fruit-and-vegetable-prices.

